# Comparison of topological, empirical and optimization-based approaches for locating quality detection points in water distribution networks

**DOI:** 10.1007/s11356-020-10519-3

**Published:** 2020-08-26

**Authors:** Giovanni Francesco Santonastaso, Armando Di Nardo, Enrico Creaco, Dino Musmarra, Roberto Greco

**Affiliations:** 1 Dipartimento di Ingegneria, Università degli Studi della Campania “L. Vanvitelli”, Aversa, Italy; 2 Istituto Studi Complessi, CNR, Rome, Italy; 3 Dipartimento di Ingegneria Civile e Architettura, Università degli Studi di Pavia, Pavia, Italy

**Keywords:** Water quality, Water safety plan, Complex network theory, Water quality detection, Sensor positioning, Water protection

## Abstract

The positioning of quality detection points as well as the frequency of sampling is a crucial aspect for the implementation of Water Safety Plans (WSPs), which have been proposed worldwide to ensure water quality and to minimize the risk from contamination in water distribution networks (WDNs). In this regard, some international legislations and best practices about quality of drinking water suggest very fine sampling frequencies, but they do not specify where the detection points should be located in a WDN. In this paper, three different approaches, based on empiricism, optimization and topology, respectively, were applied to locate detection quality points in a WDN. The comparison highlighted that empirical approach commonly adopted by water utility practitioners is unsatisfactory. The optimization-based approach, although performing significantly better, is difficult to apply, since it requires a calibrated hydraulic model. The topological approach, based on the use of the betweenness centrality and not requiring any hydraulic information and simulation, proves to be effective, and it can be easily adopted by water utilities to identify the location for quality detection points, due to its simplicity compared with the optimization-based approach.

## Introduction

Water distribution networks (WDNs) are among the most important critical infrastructures of modern society, because their security is a priority issue for public health. They represent the final component of a more complex water system that consists of numerous distribution pipes and nodes, with many devices such as pumping stations, storage tanks, valves, etc. However, WDNs have several vulnerabilities to a large variety of threats (physical, chemical and biological), which may put the quality and safety of supplied water at risk (Mays 2004). Indeed, physical disruption of supply system (e.g. breaking of main pipes, failure of pumping station) can interrupt the service for many users, while spreading of chemical or biological agents can cause diseases or death, with a great impact on users’ health and safety.

Several authors reported real cases of contamination events that occurred in water supply systems. Gray (2008) discussed water contamination accidents in UK between 1990 and 2001 and described three examples of contamination: main pipe, treatment plant and abstraction point of raw water from river, including information about the reaction time of the Water Company, the source of incident and the remediation actions. Other authors (Winston and Leventhal 2008) reviewed two unintentional contamination events that occurred in Tel Aviv, Israel (2001) and Camelford, England (1988), which occurred by dumping, accidently, 5 tons of ammonia into the drinking water reservoir and 20 tons of aluminum sulfate into the wrong treatment plant tank, respectively. Furthermore, Xin et al. (2016) grouped typical accidental contaminations that occurred in Chinese WDNs in three categories: source contaminations, wrong or illegal connections and scarce water quality management.

In general, contamination events could be accidental (unintentional) or intentional. Accidental contamination represents a random phenomenon of drinking water pollution and can occur in different locations of the whole water system. Instead, intentional contamination is a deliberate water pollution and represents a major risk for society with serious consequences. After 11 September 2001, many countries adopted guidelines for water quality monitoring and emergency action plans (United States Environmental Protection Agency 2003, 2009; Hasan et al. 2005; Keohane and Reform. 2005). In addition, contaminations can be classified on the timespan of the event (Mays 2004): (a) short term, if the contamination occurs over a period of minutes, hours or 1 day and, as a result of the exposure to the contaminated water, disease, infection or death are recorded within a short period; (b) long term, if the contamination occurs over a period of months or years and the resulting illness could appear after several years from the initial exposure.

The growing interest in drinking water protection and the necessity to satisfy the water quality standards led to the definition of Water Safety Plans (WSPs) proposed by the World Health Organization (WHO). WSPs ensure the safety of water by means of a comprehensive risk assessment and management for all phases of the water system (Davidson et al. 2005). One of the main steps to develop a WSP is to define a monitoring system for the distribution of drinking water. Indeed, an online contaminant monitoring system represents the main countermeasure to reduce the likelihood of both intentional and unintentional contaminations (ASCE 2004; Roberson and Morley 2005). This system should be able to early detect contamination events and promptly provide information about the entry point of contaminant into the distribution system. When a contamination accident is identified, three main actions should be performed to minimize the contaminant spread into water supply system: (1) alert users not to use the contaminated water, (2) isolate the hydraulic sector of the network to limit health risks and (3) remove the contaminant (Di Nardo et al. 2014).

However, the development of monitoring systems still represents a challenge not only for water utilities but also for researchers, because the definition of the number and positioning of quality detection points is not trivial, as well as the selection of water quality parameters to be monitored. As is known, indeed, the detection of contaminant intrusion into a WDN is a difficult issue, due to the huge number of possible scenarios depending on different source locations, intrusion times, contaminant substances, etc. Therefore, the placement of water quality detection points represents an “open problem” for the scientific community, with the aim of designing a reliable and cost-effective sensor distribution, crucial for protecting the users from the effects of water contamination (Giudicianni et al. 2020).

Traditionally, in the water sector, the positioning of quality detection points in water distribution networks was treated empirically, starting from some simple assumptions to localize detection points, mainly in more densely urbanized areas or in pre-existing fountains.

In the two most recent decades, the problem of sensor placement has been faced by the scientific community as a single or multiobjective optimization problem, coupled with hydraulic and quality simulations. Thus, it is possible to define different strategies of sensor placement based on the following features: (a) need of hydraulic and quality simulation, (b) use of optimization methodology and (c) known/unknown maximum number of sensors (Rathi and Gupta 2014). With regards to the optimization solver, heuristic and evolutionary algorithms are widely employed and the main used objective functions are essentially (Di Nardo et al. 2014; Tinelli et al. 2017): number of individuals exposed to a contaminant; number of detected contamination events; length of contaminated pipes; amount of contaminant consumed by users; detection time. In order to investigate the effectiveness of optimization techniques to find the optimal solution for the placement of quality sensors or detection points, the Battle of the Water Sensor Networks (BWSN) was undertaken (Ostfeld et al. 2008), highlighting that no general “optimality criteria” can be found, but some proposed methodologies can define a good monitoring layout of solutions. Anyway, it is worth highlighting that all these procedures require hydraulic simulations to be carried out on a calibrated WDN model.

Some methodologies have recently been developed, which do not require a hydraulic or quality simulation. Therefore, they can be applied even when no numerical model is available for the WDN. In this regard, the work (Davis et al. 2014) provided a framework to assess the consequences of contamination events in the absence of a detailed network model.

Recently, other approaches were developed based on complex network theory (Boccaletti et al. 2006), relying on the knowledge of the topological structure of the networks. Nazempour et al. (2018) coupled an optimization algorithm with the complex network theory to solve the problem of sensor placement. Di Nardo et al. (2018) proposed the application of Graph Spectral Techniques (GSTs) to identify the most important nodes in which locating water quality detection points or sensors. Giudicianni et al. (2020) proposed a topological placement of quality sensors in WDNs without resorting to hydraulic modeling. Other authors employed clustering analysis in order to identify the location of monitoring points in a residential WDN (Delpla et al. 2018). These challenging tasks can be approached only through network topological data, even when no other hydraulic information is available.

Though European law 98/83/CE (European Comission 1998) and Italian Legislative Decree 31/2001 suggest only a minimum number of samplings based on total delivered water volume without giving indications on the positioning of the monitoring points; this paper compares three different approaches with the aim of providing an effective and simple method to identify a set of nodes where quality detection points or sensors can be located: (1) an empirical approach, generally adopted by Water Utility to comply with legal requirements; (2) an optimization-based approach, proposed by Sandia National Laboratories in the Chama framework (Klise et al. 2017), based on the linear programming; (3) a topological approach.

### Empirical approach

The first approach provides the placement of quality detection points in WDNs according to some empirical criterions: area with high number of supplied inhabitants, network elements where water characteristics could change throughout the year, terminal WDN pipes where contamination events can occur and critical WDN points where a failure directly or indirectly causes alteration of delivered water quality.

Sampling along the WDN can be easily performed by means of public fountains, taps installed upstream from the household flow meter, local tanks that supply water directly into the network, tap of public buildings or private houses.

Clearly, all these criteria are empirical and require only network topology and some geographical information, technical expertise and know-how on the water system. No hydraulic data (as flow, diameters, flows, etc.), no simulation and calibration processes or optimization procedures are required.

### Optimization-based approach

In the two most recent decades, many authors proposed different approaches to optimize the positioning of quality detection points based on different algorithms and procedures, as reported in Hart and Murray (2010) and Adedoja et al. (2018), who implement both single or multiobjective sensor placement models. The implemented objective functions are related to expected contaminated water volume, detection likelihood, detection time and exposed population. These optimization problems have NP-hard complexity (nondeterministic polynomial-time hard, Wang 2013) because the optimal sensor placement in a network is a combinatorial problem, as proven by Xu et al. (2013), if all possible scenarios are investigated. Consequently, the search of optimal solution is often computationally extremely burdensome.

As a result, recently, the Sandia National Laboratories developed the Chama software (Klise et al. 2017) in Python package. This software represents an international benchmark for sensor placement optimization in a wide range of applications. Chama includes mixed-integer, stochastic programming formulations to provide an optimal positioning of quality detection points, and it allows the user to select the technology of sensors in a monitoring system. It is possible to define four types of sensors: stationary and static point sensors, stationary and static cameras. Furthermore, sensors can monitor continuously or at defined sampling times.

In this study, the *P*-median formulation is applied to optimize the position of a pre-assigned number of quality detection points to be installed in the WDN, with the objective of maximizing the number of detected events. The *P*-median problem lies in finding the location of *P* facilities on a network in order to minimize a cost function. The main advantage of this formulation is that it can be solved in polynomial time with the number of nodes for fixed values of *P* (Daskin 1997). In particular, Berry et al. (2008) and Watson et al. (2009) proposed the *P*-median formulation to define sensor location in water supply networks. In this case, the *P*-median formulation consists of minimizing the objective function of Eq. (1) subject to constraints of Eq. (2–6):

1 $$ \operatorname{minimize}\ {\sum}_{\mathrm{a}\in \mathrm{A}}{\alpha}_{\mathrm{a}}\ {\sum}_{i\in {\mathcal{L}}_{\mathrm{a}}}{d}_{\mathrm{a}\mathrm{i}}{x}_{\mathrm{a}\mathrm{i}} $$

2 $$ \mathrm{subject}\ \mathrm{to}{\sum}_{\mathrm{i}\in {\mathcal{L}}_{\mathrm{a}}}{x}_{\mathrm{a}\mathrm{i}}=1\forall a\in A $$

3 $$ {x}_{\mathrm{ai}}\le {s}_{\mathrm{i}}\forall a\in A,i\in {\mathcal{L}}_a $$

4 $$ \sum \limits_{\mathrm{i}\in \mathrm{L}}{c}_{\mathrm{i}}{s}_{\mathrm{i}}\le p $$

5 $$ {s}_{\mathrm{i}}\in \left\{0,1\right\}\kern0.5em \forall i\in L $$

6 $$ 0\le {x}_{\mathrm{a}\mathrm{i}}\le 1\forall a\in A,i\in {\mathcal{L}}_{\mathrm{a}} $$

where *A* is the set of contamination events; ${\mathcal{L}}_{\mathrm{a}}$ is the set of quality detection points able to detect the contamination event *a*; *L* is the set of all candidate detection points; *α*_a_ is the probability of occurrence of the contamination event *a*. As the first attempt, all scenarios can be assumed equiprobable with probability computed as 1/*dim*(*A*); *d*_ai_ represents the value of impact measure or damage metric of event *a* first detected by a sensor *i*; *x*_ai_ (Eq. (6)) is a continuous variable between 0 and 1 and equal to 1 if the sensor *i* is the first to detect the contamination event *a*; *s*_i_ is equal to 1 if sensor *i* is selected and 0 otherwise; *c*_i_ is the cost of sensor *i*; *p* is the available budget to install quality detection points, in this study representing the total number of quality detection points to be installed in the network with the same cost *c*_i_ = 1.

Constraints of Eqs. (2) and (3) ensure that the contamination event *a* ∈ *A* is detected only by one sensor and that the sensor *i* is being selected by the optimization procedure, respectively. Indeed, the constraint of Eq. (4) ensures that cost of quality detection points or sensors is lower than available budget or the number of quality detection points or sensors to be placed in network is equal to *p* by assigning *c*_i_ = 1 to each sensor.

In this study, Chama framework is applied to minimize the number of exposed user *N*_eu_ with a pre-assigned number of quality detection points to be defined for the WDN. This choice is due to the main task of a sampling plan for water quality monitoring, European law 98/83/CE, that is to identify as many contamination accidents as possible reducing the number of exposed users. Hence, the contamination scenarios were generated using EPANET 2.0 software (Rossman 2000).

This choice of objective function, for the optimization with Chama tool, is in compliance with the objective that a water utility can consider during the design of localization of detection points, as illustrated in the previous section.

### Topological approach

As known, WDNs can be represented as a graph *G = (V,E)*, with *V* and *E* representing the set of *n* vertices or nodes and the set of *m* pipes or edges, respectively, and can be studied as a complex network. Therefore, several metrics from complex network theory can be used to evaluate the behavior of WDNs (Yazdani and Jeffrey 2010; Giudicianni et al. 2018).

The identification of the most influential spreading nodes in a complex network plays a key role to control and understand a complex system, such as social networks, World Wide Web, etc. Recent works have shown that different centrality metrics are able to discover the spreading capabilities of a node with different levels of accuracy (de Arruda et al. 2014). This opportunity suggests applying centrality metrics to locate sampling points or monitoring stations in WDN, when no data are available for hydraulic simulation models or for network calibration. According to the approach proposed by Di Nardo et al. (2018), the sensor placement can be obtained following three steps:

- cluster WDN in *k* subsets of nodes with *k* the desired number of quality detection points to place in the network;
- for each cluster, rank nodes according to the score attributed by the corresponding values of the selected centrality metric
- define the most influential nodes belonging to *k*-th subset, where quality detection points must be placed.

The original approach of Di Nardo et al. (2018) suggested the principal eigenvector (Newman and Newman 2010) as centrality metric and spectral clustering (Shi and Malik 2000) to define the *k* subsets of nodes. Differently, in this paper, betweenness centrality (Linton C. Freeman 1977), originally adopted to study the importance of an individual in a social network (Boccaletti et al. 2006), is used to define the locations of monitoring points in the WDN. In a network, a node is central if it falls along the shortest path between pairs of other nodes (Linton C. Freeman 1977), and from this point of view, the betweenness *g(v)* of node *v* can be computed as follows:

7 $$ g(v)={\sum}_{\mathrm{s},\mathrm{t}\in \mathrm{Vs}\ne \mathrm{t}}\frac{\sigma_{\mathrm{s}\mathrm{t}}(v)}{\sigma_{\mathrm{s}\mathrm{t}}} $$

where *σ*_st_ is the number of shortest paths that connect nodes *s* and *t* and *σ*_st_(*v*) is the number of shortest paths that link *s* and *t* while passing through node *v*.

In this work, betweenness was chosen as centrality metric because it measures the importance of a node not only in terms of adjacent nodes but in relation to the whole network (Scott 2000). For example, a node with a low degree of connections results central for the network if link nodes that otherwise are isolated. Although there is not a direct relationship between the applied metric and the hydraulics of a WDN, betweenness catches well the topological behavior of the water systems providing an effective way for the positioning sensors according to higher value of betweenness involves choosing nodes traversed by a greater number of shortest paths. Therefore, it facilitates contaminant detection.

Other authors (Yoo et al. 2015) have applied betweenness centrality to locate water quality sensors in water distribution network but their methodology applies results of hydraulic simulations as input. On the contrary, the proposed topological approach does not need any hydraulic simulation, it requires very low computation time and it is very simple to implement for water utilities.

### Case study and results

Events of accidental contamination may occur in several ways in a WDN: various contaminants may enter at different concentrations in one or more points of the WDN. Indeed, the problem of detecting an intentional contamination, such as a terroristic attack or the deliberate poisoning of drinking water, would require a high sampling frequency to be used and chemical analyses to be carried out in real time, which is not in compliance with the actual international laws and best practices. Clearly, in an unintentional contamination, the negative effects on users’ health, in a short-term period, can be considered lower than for an intentional contamination. However, a significant delay can occur between water sampling and contaminant detection, entailing that users may ingest a great amount of polluted water before detection and subsequent interruption of supply.

In this paper, in order to define the characteristics of the accidental contamination event, a private well has been considered as the contaminant source, from where pollutants enter the WDN. Indeed, a low pressure in the network, due either to uncommonly high water demand or to failures and water scarcity in the system, may cause the pressure exerted by a pump, extracting groundwater from a contaminated private well used as a supplementary water resource, to exceed the pressure in the network pipes, resulting in ingress of well water into the WDN (Kroll 2006). In this case, a contaminant is introduced into the water distribution system. Arsenic (As) is chosen as pollutant, because high concentrations of natural origin are sometimes found in groundwater in volcanic areas (International Agency for Research on Cancer (IARC) 2004), as is the case with the analyzed case study.

Arsenic has no odor or taste, and only analytical tests can detect its presence. Having no evidence of contamination, users cannot feel the danger and, as a result, cannot alert the authorities or the water utility. Moreover, if the operating system of water quality monitoring fails to discover arsenic, the contamination may cause heavy effects on human health: a long-term exposure to arsenic can lead to stomach pain, vomiting, diarrhea, impaired nerve function, and skin cancer (International Agency for Research on Cancer (IARC) 2004). Hence, over the years, the limit of arsenic concentration in drinking water has been fixed lower than 10 μg/L (European Comission 1998) to reduce health risk (Achene et al. 2010). To simulate a contamination accident able to generate a concentration value in the network at least equal to the regulatory limit, a total mass of arsenic, equal to 0.5 kg, is injected in the network by the contaminated well during the hour of peak water request.

**Fig. 1:**
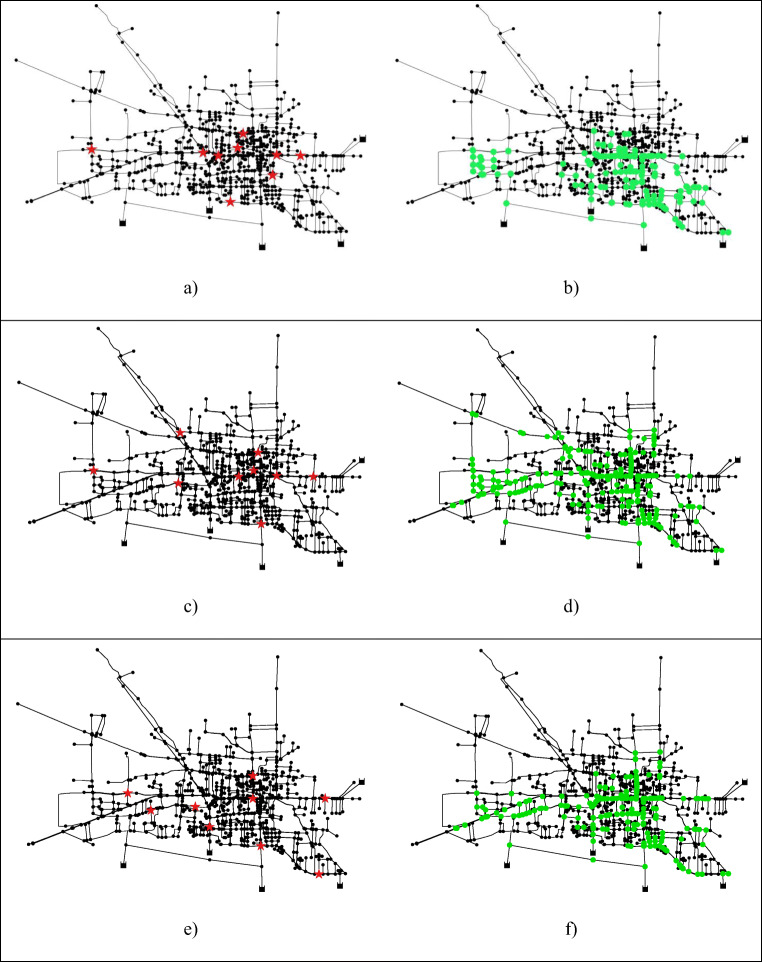
WDN of Giugliano in Campania with the representation of monitoring points according to the different compared approaches (**a**, **c** and **e**) and detected injection nodes (**b**, **d** and **f**)

The considered case study is the WDN of Giugliano in Campania (Fig. 1), a city of Southern Italy with about 120,000 inhabitants. The network depicted in Fig. 1 supplies the city centre populated about by 70,000 inhabitants. It has 994 demanding nodes, 5 source nodes with assigned head and 1077 pipes. In the typical service day, the water demand adds up to 15,213 m^3^. For this case study, *N*_s_ = 994 scenarios of contamination events are evaluated under the assumption that:

- contaminant injection may arise in one node at a time with the same probability of occurrence;
- contamination begins at 11:00 am and continues for 1 h.

Admittedly, in real world, contamination can occur at any time causing a different contaminant spread according to pipe velocity and flow directions. The hypothesis that contamination starts at 11:00 am and ends at 12:00 am allows analyzing contaminant spreading during the peak hour which represents the worst scenario for the considered case study.

For each scenario, hydraulic and quality simulations are carried out by using the EPANET 2.0 software. The current monitoring plan for the WDN of Giugliano, as provided by Municipality, identified 9 detection points where water samples are located. Therefore, in order to compare the results, the same number of detection points was assumed also using centrality metrics and Chama optimization algorithm.

In Fig. 1, the red symbols represent sampling points according to the current monitoring plan obtained with empirical approach (Fig. 1a), to the optimization approach with Chama software (Fig. 1c) and to topological approach with betweenness centrality (Fig. 1e). Since the *i-th* scenario represents a contamination event that occurs in *i-th* node, the detected events can be localized on the network by highlighting the injection node; in this way, the green nodes in Fig. 1 b, d, and f represent scenarios successfully detected by current monitoring plan, Chama software, and topological approach, respectively. As is clear from the figures, Chama software covers contaminations originating from a wide area of the WDN, but not from the peripherical area, while empirical and topological approaches cover only contaminant propagating from a small area around the city center.

The following indices are applied to quantitatively compare the performance of the different approaches used to locate the sampling points: the number of detected scenarios *N*_ds_, the percentage of identified scenarios *P*_det_ = *N*_ds_/*N*_s_ × 100, the number of exposed users *N*_eu_, and the detection time *t*_d_ as the minimum detection time among all defined quality detection points (the detection time is computed as the elapsed time from the start of the contamination event to the first identified presence of a contaminant by the sensor). In Table 1, the mean and maximum values of each index are reported.

**Table 1:** Number of detected scenarios (*N*_ds_), percentage of identified scenarios (*P*_det_), mean and max of exposed users (*N*_eu_) and detection time (*t*_d_*)* computed for empirical, optimization and topological approaches

	*N*_ds_	*P*_det_	*N*_eu_		*t*_d_ [min]	
			Mean	Max	Mean	Max
Empirical approach	185	18%	1258	11,930	68	355
Optimization approach	285	29%	721	5253	62	405
Topological approach	245	25%	1337	10,002	72	435

The current monitoring plan adopted by municipality, based on empirical approach without any hydraulic simulation, is able to detect *N*_ds_ = 185 scenarios (*P*_det_ = 18%). The detection points defined according to topological approach with the computation of betweenness centrality, without any hydraulic simulation, detect 245 (*P*_det_ = 25%) contamination events. Finally, using the approach based on Chama package for sensor placement optimization, detection points are able to identify *N*_ds_ = 285 contamination events (*P*_det_ = 29%).

As expected, the positioning of detection points obtained with the heuristic optimization procedure allows reducing considerably the number of exposed users (also because the minimized objective function was chosen exactly equal to the number of exposed users). However, the innovative approach, based only on topological information without hydraulic simulations, proves to have a similar effectiveness.

**Fig. 2:**
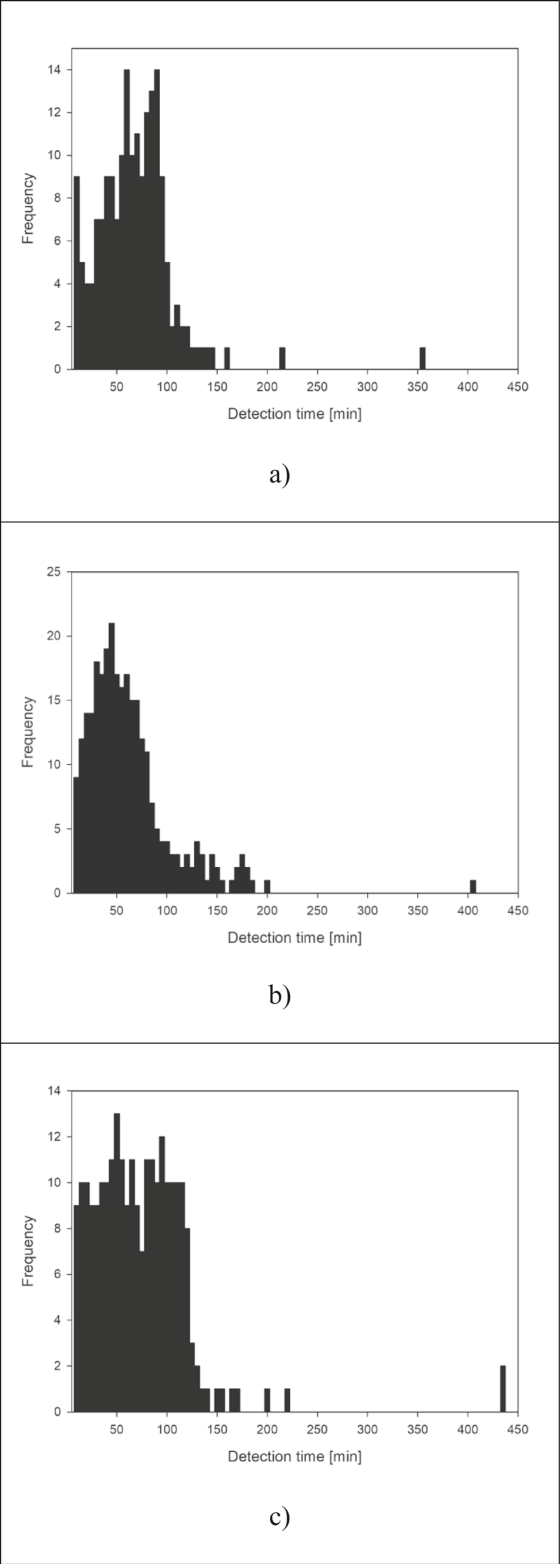
Histograms of detection times computed for **a** current monitoring plan, **b** monitoring points provided by Chama package and **c** betweenness centrality

Additionally, histograms of detection time for all the tested approaches are reported in Fig. 2. The average minimum detection time is about 62 min for the layout of detection points proposed by Chama framework and the maximum is about 435 min for the topological approach (see also Table 1).

The mean detection time computed for Chama is lower than both empirical and topological approaches, despite it detects 12 scenarios 150 min after the beginning of contamination. The current monitoring plan and the topological approach, instead, detect 3 and 7 scenarios, respectively, after 150 min.

The average detection time computed for the layout of monitoring points proposed by the topological approach is about 72 min. It is worth remarking that the average detection time of the current empirical monitoring plan is quite similar to that computed for sensor placement proposed by betweenness centrality, although the centrality metric is able to detect a greater number of contamination events compared with the current monitoring plane.

**Fig. 3:**
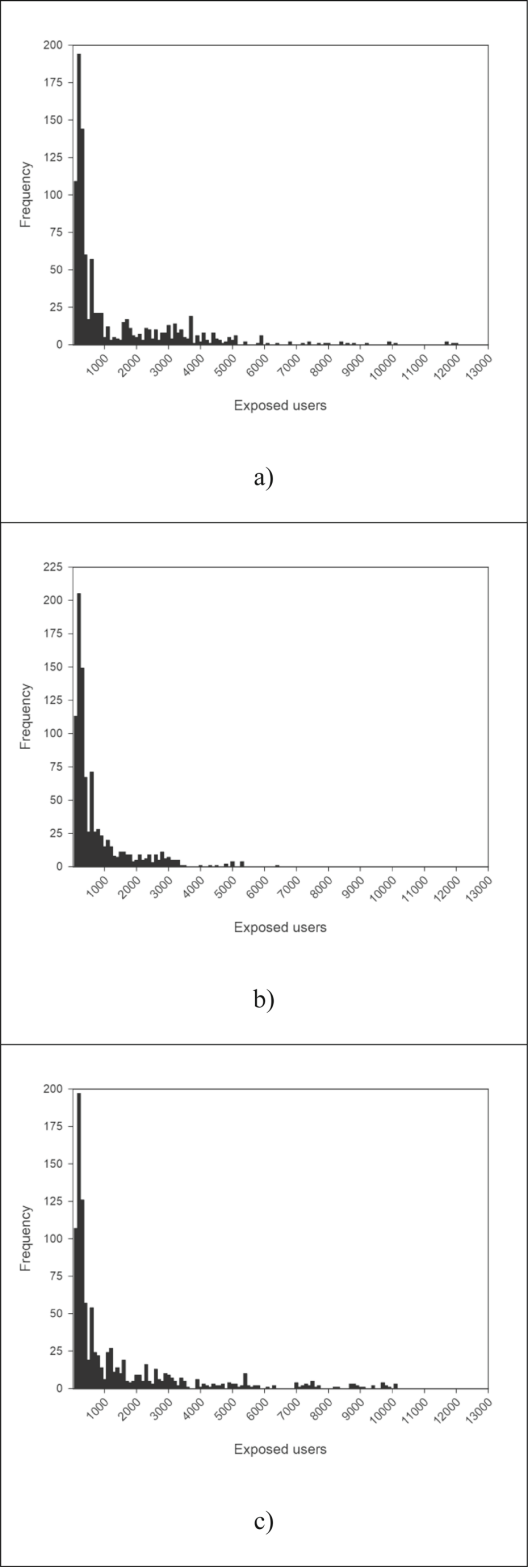
Histograms of number of exposed users for **a** empirical approach (current monitoring plan), **b** optimization approach (by Chama package), **c** topological approach (with betweenness centrality)

Figure 3 illustrates histograms of contamination impacts in terms of exposed users computed for all monitoring points provided by the adopted approached.

The distribution of contamination impacts of current monitoring plane has mean and maximum values equal to 1258 and 11,930 individuals, respectively, with many scenarios that yield high consequences: 74 events produce a number of exposed users greater than 3500. It is worth remarking that the maximum value of the distribution reported in Fig. 3a is equal to 17% of the total population. Despite a low value of the percentage of identified scenarios (*P*_det_ = 29%), the layout of detection points proposed by Chama strongly limits the consequences for customers. Specifically, only 14 scenarios produce more than 3500 exposed users. In this case, the distribution of impacts, Fig. 3b, shows a mean equal to 721 individuals and a maximum equal to 5253, about 8% of total population.

Conversely, the average and maximum values of impact distributions computed for the detection points provided by the topological approach (Fig. 3c) are 1337 individuals and 10,002 individuals (about 2% and 14% of total population), respectively. The average value of impact distribution of the topological approach is greater than Chama as well as the number of events that produces an impact greater than 3500 (101 scenarios contaminate more than 5% of total supplied population). In terms of contamination impact, the solution provided by Chama approach performs better than both the empirical and topological approaches, because it was identified by minimizing the number of exposed users. However, the application of the topological approach allows obtaining good results compared with the current empirical monitoring plan in terms of detected events and contamination impact.

These results highlight that the topological approach can be a valid option to identify monitoring points in a WDN compared with the current monitoring plane based only on expert knowledge.

However, it is worth highlighting that the results show that, in all the investigated approaches, the number of 9 detection points defined by the municipality for the WSP of the WDN of Giugliano is inadequate to monitor all possible contamination events. Indeed, even the optimization approach is able to identify a small number of contamination events, with a *P*_det_ < 50%.

In order to better investigate this point and evaluate how many quality detection points are required to increase the effectiveness of detection system, the exposed users and identified contamination scenarios are studied as a function of the number of detection points.

**Fig. 4:**
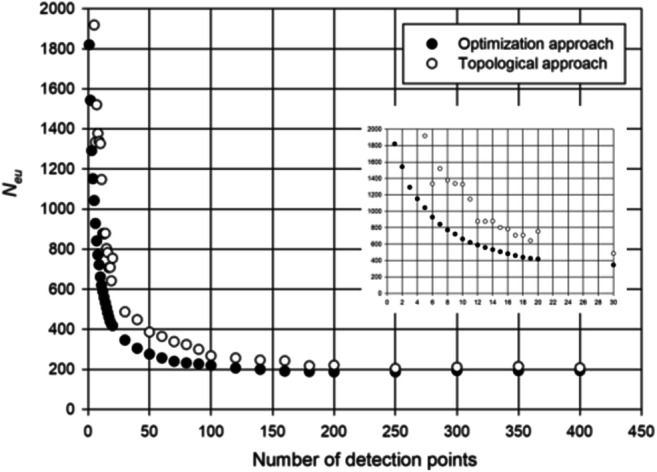
Number of detected events (*N*_eu_) computed with optimization approach (black points) and topological approach (white points) varying the number of monitoring points

Figure 4 plots *N*_eu_ vs number of detection points for the optimization-based and topological approaches. As expected, this figure clearly shows that Chama provides better results in terms of *N*_eu_ up to 100 detection points, while beyond that limit, the results become almost identical.

Anyway, Fig. 4 shows the effectiveness of both approaches, which are able to improve significantly the contamination detection when the number of installed detection point increases from 1 to 10. In fact, the number of exposed users *N*_eu_ is reduced from about 1800 to about 600 with the Chama software, and from about 1900 to 1300 with the topological approach. A further analysis of the results shows that significative improvements, for the case study, can be obtained adding other 10 detection points with a decrease down to 400 and 700 exposed users, respectively, with the optimization-based and topological approach. Therefore, for the case study, a number between 10 and 20 detection points can represent a good trade-off in economic terms.

**Fig. 5:**
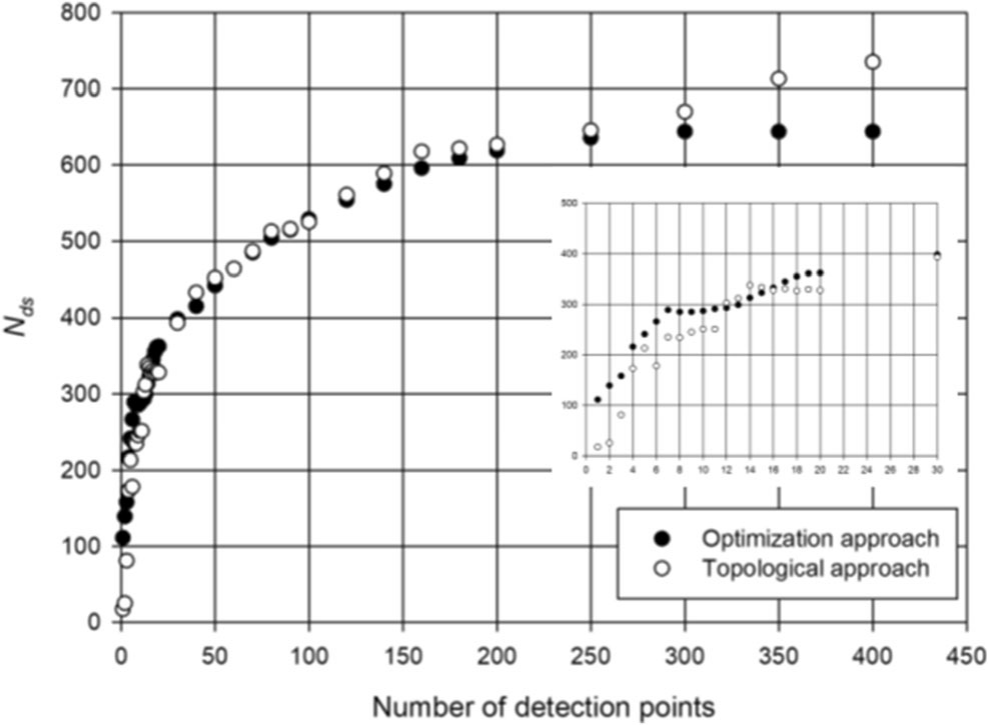
Number of detected scenarios (*N*_ds_) computed with optimization approach (back points) and topological approach (white points) while varying the number of monitoring points

In Fig. 5, the relationships between detected scenarios *N*_ds_ and number of detection points are reported. They show that more than 400 monitoring points are needed to cover all possible scenarios both with the Chama and topological approach. Evidently, this result reveals that the problem is very difficult to be faced in an exhaustive way, because it is extremely expensive to locate a large number of detection points and only sub-optimal solutions can be achieved. It is worthy to note that with a large number of detection points, topological approach can perform better than Chama in terms of detected scenarios.

Furthermore, the results of Fig. 5 also show that, for the case study, a number between 10 and 20 monitoring points can represent a good trade-off in economic terms; significant further improvement can be observed with more than 20 monitoring points both with Chama and topological approach but they are not economically sustainable.

Finally, it is worth highlighting that the number of 9 monitoring points chosen by the Italian water utility for the case study of Giugliano in Campania can represent the minimum reasonable number of detection points for achieving a suitable level of protection.

## Conclusions

This work allowed comparing the effectiveness of different methods for choosing a potential set of locations where to choose quality detection points in WDNs to mitigate the contamination risk according to Water Safety Plans.

The study reveals that the empirical approach, based only on simple operative suggestions, provides a low degree of protection, with a percentage of detection lower than 20%, even if it is applicable without any hydraulic information on the WDN.

Although the results of Chama simulations are encouraging, it requires a well-calibrated hydraulic model which, in many cases and in many countries, is difficult to obtain. Instead, the proposed topological approach improves the performance of the empirical method and, at the same time, guarantees similar effectiveness to the optimization-based approach. Furthermore, this method does not require any hydraulic simulations but only knowledge of the network topology. Therefore, it is simple to learn and quick to apply for water utility operators.

The case study of Giugliano in Campania showed promising results in terms of detected contamination events, detection time, and contaminated population.

In addition, the adopted centrality metric can be modified by weighting pipes of network by using hydraulic or geometrical data, e.g., diameter and length of pipes or number of users supplied by each node.

Another important result of the paper is that, even when the most efficient location of quality detection points is identified through an optimization approach, the installation of all the sensors required to identify all possible contamination scenarios, and thus reducing to zero the number exposed users, is economically unfeasible. In the analyzed case study, it is clear that a good economic and technical compromise can be achieved with percentages equal to 35–50% with a number of monitoring sensors between 10 and 20.

In the future, the effectiveness of other metrics will be investigated, also using proper weights, for the topological approaches in order to improve the proposed methodology for water quality monitoring.
